# Postural correlates of pleasant landscapes visual perception

**DOI:** 10.3389/fpsyg.2025.1527691

**Published:** 2025-02-05

**Authors:** Mbarka Akounach, Thierry Lelard, Harold Mouras

**Affiliations:** ^1^UR-UPJV 4559, Functional Neurosciences Laboratory, Health Research University Center, Medicine Department, University of Picardy Jules Verne, Amiens, France; ^2^UR-UPJV 3300, Physiological Adaptations to Exercise and Exercise Rehabilitation, Sport Sciences Department, University of Picardy Jules Verne, Amiens, France

**Keywords:** pleasant landscapes, environmental perception, postural modulation, mental simulation, embodiment

## Abstract

**Introduction:**

The interplay between motor skills and emotions in the brain represents a significant and longstanding research question. Recently, posturography has provided new insights into this subject. Understanding the biological processes that influence the appreciation of nature and landscapes is also a crucial concern, prompting various experimental methods and theoretical frameworks. This research aimed to propose, for the first time, the use of posturography to study the different ways postural control is modulated by visual perception of pleasant scenes.

**Methods:**

A total of 37 participants (27 females, 10 males; mean age = 24 years ±5 years) were shown images of pleasant and neutral landscapes, while posturographic data were collected. Two viewing conditions were employed: *passive* vision and *active* vision, where participants were instructed to envision themselves in the presented scenes.

**Results:**

The results indicated a differential modulation of the postural response based on valence factors (pleasant vs. neutral) and mental simulation (passive vs. active). Notably, significant differences in approach-avoidance behavior were observed approximately 7 s after the onset of stimulus viewing.

**Discussion:**

The findings are discussed in relation to major theories in environmental psychology, highlighting the central role of emotional and embodiment processes in appreciating pleasant environmental scenes and related motor behaviors.

## 1 Introduction

Since the pioneering work of Darwin (1872), the question of the interaction between motor and emotional processes has long been central to science. In particular, emotional stimuli can elicit automatic, instinctive responses that allow for rapid behavioral adaptations (Mauss et al., 2007; Brosch et al., 2010; Carretié, 2014). With regard to the experimental study of this interaction, in the last ten years, an interesting method for studying the interaction between emotion and motor skills has emerged in the last decade: the study of the modulation of postural control by socioaffective information processing (Lelard et al., 2019). To explore the different results obtained and to understand the complexity of the postural response, the interest in studying its temporal dynamics in detail has been mentioned (Mouras and Lelard, 2018; Lelard et al., 2017). In recent years, this methodology has been successfully applied, for example, in the functional context of empathy for pain for example (Mouras and Lelard, 2021). Interestingly, the same methodology has also been applied to the scientific study of more “societal” issues, such as pollution visual perception (Akounach et al., 2022), demonstrating the differential modulation of posture by (i) the *valence* of the stimulus (painful vs. non-painful in the model of empathy for pain, but more importantly by its emotional valence) and (ii) the intensity of the participant’s *immersion* in the depicted scene.

The central nervous system is primarily responsible for maintaining the body’s homeostasis. To achieve this, it measures and interprets information from the internal and external environment and responds to significant variations in measured values by initiating adaptive behavioral responses, particularly motor responses (Clarke and Harris, 2004; Murray and Sessle, 2024). Consequently, the study of the psychological and physiological processes (both peripheral and central) involved in the perception of natural environments and the associated behavioral responses has emerged as a major contemporary research question in cognitive neuroscience (Kaplan and Kaplan, 1989; Berman et al., 2021; Kühn and Gallinat, 2024).

Shortly before the advent of brain imaging techniques, theoretical proposals for the articulation of these processes were put forward. The interaction between motor and emotional processes is central to these proposals. *Affordance* is an interesting concept that proposes integrating into the perceptual processes of a scene or object, its use, its practical aspect, i.e., aspects other than those purely related to its basic visual characteristics. In landscape perception, this concept has led some authors to propose, for example, the notion of processual landscape (Menatti and Casado da Rocha, 2016). In our framework, *biophilia* is also of great importance, postulating an automatic human tendency to approach nature (Schiebel et al., 2022), thus implicitly linking motor and emotional processes (Gaekwad et al., 2022). Regarding the mechanisms involved, several authors have suggested that the ability to empathize with nature is central to the human relationship with nature (Schultz, 2001; Tam, 2013; Geiger et al., 2017).

As a result, numerous studies have attempted to characterize, at various levels, what might be termed “biomarkers” of the perception of environmental scenes and landscapes. For the purposes of our study, we will focus exclusively on work that deals with the visual perception of environmental scenes or landscapes. According to *ElectroEncephaloGraphy* research *(EEG)*, posterior alpha power may serve as a biomarker for differences associated with exposure to natural environments (Hopman et al., 2020). In addition, increased frontal alpha asymmetry has been observed in response to landscape stimuli (Mavros et al., 2022). Kaiser (2022a) reported a reflection of the attractiveness of natural scenes in the EEG signal within 200 ms (i.e., during the perceptual process). Kaiser (2022b) found that alpha and beta frequency bands are modulated by the aesthetic perception of nature. *Eye-tracking* has proven to be an effective experimental method for investigating these biomarkers allowing to show that panoramic pictures generate more fixations, while features such as the degree of openness and heterogeneity influence viewing patterns (Schirpke et al., 2022) and that natural landscapes are often perceived as more attractive than urban landscapes (Dupont et al., 2014). *Functional Magnetic Resonance Imaging* (*fMRI*) has also contributed to our understanding of the brain areas involved in processing environmental scenes mainly with:

- Few of the available studies report motor-related activations in brain areas that are commonly interpreted to be associated with motor processes. Dadvand et al. (2016) found that increased green space exposure was associated with increased grey matter volume in prefrontal cortices and the left premotor cortex and increased white matter volume in the right prefrontal cortex, left premotor cortex, and both cerebellar hemispheres, suggesting an effect of prolonged green space exposure on brain plasticity and cognition. Pati et al. (2014) reported brain activations in response to sky composition images related to spatial cognition and motion/motor balance (internal sense of motion). Zhao et al. (2020) found overlapping neural networks involved in the aesthetic judgment of static and dynamic landscapes, but an increase in visual motion-related areas for dynamic landscapes. Di Dio et al. (2007) reported activations in motor cortex areas during aesthetic evaluation of human subjects and natural scenes, underlying the role of motion perception in aesthetic judgment;
- Most importantly, most fMRI studies of landscapes/natural environments reported activations in emotional areas. Chang et al. (2021) reported a key role of the posterior cingulate cortex in the stress response benefits of viewing green landscapes. Isik and Vessel (2021) showed modulation by the aesthetic appeal of natural landscape films in specific regions adjacent to scene and motion processing areas. They argue for a neural local transformation from a feature-based visual representation to an “elemental affect” representation. Kim et al. (2010) reported differential emotional activations in response to natural and urban landscapes.

An important question is also the “way” in which the environmental scene is observed. *Passive* observation alone does not fully capture the complexity of landscape perception. Our previous studies have demonstrated the importance of what we call *simulation* or *embodiment*. The observation of environmental scenes is sensitive to the effect of instructions or immersion, as shown in previous studies (Akounach et al., 2022; Beaumont et al., 2021). Indeed, the level of engagement and the context in which participants visualize landscapes can significantly alter their perceptual experience and neural responses. This highlights the importance of considering not only *what* is observed, but also *how* it is observed and the mental state of the observer. The interplay between passive observation and active engagement provides a more comprehensive understanding of landscape perception and its underlying neural mechanisms.

While eye tracking, EEG and fMRI have been used extensively to study landscape perception, posturography has not been used to study pleasant landscapes perception. Given the importance of immersion and motor processes in landscape perception, our aim was to investigate postural responses and modulations in the perception of pleasant environments in parallel with physiological responses. We wanted to shed light on the motor correlates of visual perception of landscapes, an aspect that has been largely overlooked in previous studies. This approach could be considered as pioneering in the study of motor correlates involved in visual landscape perception. In this way, we hope to gain a more comprehensive understanding of how humans *interact with* and *respond to* pleasant environmental stimuli at both cognitive and motor levels. We expected to observe a positive correlation between the perceived pleasantness of landscapes and the magnitude of forward postural sway, indicating an unconscious desire to approach or “enter” the pleasant scene. With regard to physiological responses (i.e., electrodermal activity), we expected differential modulation by both valence (pleasant vs. non-pleasant) and viewing condition (passive vs. active).

## 2 Materials and methods

### 2.1 Participants

The study included 37 participants (27 females, 10 males; mean age = 24 years ±5 years) with no history of neurological, psychiatric, or oculomotor disorders. All participants provided written informed consent before their involvement. The study protocol was approved by the Comité d’Ethique pour les Recherches Non Interventionnelles (CERNI, n° 2024–43, Université de Picardie Jules Verne, Amiens, France) and conducted in accordance with the déclaration of Helsinki (World Medical Association, 2013). To determine the required sample size for our study using the Wilcoxon signed-rank test, we conducted a power analysis in R. We set the significance level (*α*) at 0.05 and aimed for a power of 0.8, estimating a small effect size of 0.2. The analysis indicated that we would need approximately 33 participants per group. We increased this number by 10% to account for potential dropouts, resulting in a final target of 36 participants to ensure adequate statistical power for detecting significant effects.

### 2.2 Stimuli selection

We conducted a pilot study where 80 images were evaluated by 27 subjects (17 females, 10 males; mean age = 27 years ±6 years). One subject was excluded due to overly repetitive and inconsistent responses. The images were grouped into two parts: 40 Landscape pictures and 40 pictures of their Neutral pairs. The visual material was in color and displayed on a 25-inch screen. The initial database was constructed from images provided by ADEME (The Ecological Transition Agency), a partial funder of the thesis. Another portion of the images was downloaded from royalty-free websites. A final portion was captured in Amiens by the team to seek a neutrality criterion. A Python script was designed to harmonize the dimensions (1,600*1200 px) and characteristics such as brightness and sharpness of pictures to ensure consistency.

The order of viewed images was randomized. A Python script was written to calculate the average ratings for each image and each subject.

Each image was presented for 3 s. For each image, participants were asked to give their subjective impression on Likert-type scales on the dimensions of *valence* (from 1 “very negative/unpleasant” to 9 “very positive/pleasant”), *intensity* (from 1 “not intense at all” to 9 “extremely intense”), and *degree of desire to initiate approach-avoidance behavior* (from 1 “strong avoidance” to 9 “strong approach”). A brief debriefing allowed for gathering general impressions of the participant about the experience, particularly in terms of emotional response.

#### 2.2.1 Stimuli validation

A repeated measures ANOVA was conducted to assess differences between Neutral and Pleasant Landscape images across various dimensions, including pleasure, approach tendency, and intensity. The analysis revealed significant differences between the conditions for all dimensions: pleasure [*F*(1,24) = 300.5, *p* < 0.001, η2 = 0.73], approach tendency [*F*(1,24) = 252, *p* < 0.001, η2 = 0], and intensity [*F*(1,24) = 59.56, *p* < 0.001, η2 = 0.56]. These results demonstrate a strong statistical distinction between Neutral and Pleasant Landscape conditions across all evaluated dimensions, with participants consistently providing higher ratings for Pleasant Landscape images.

Our objective was then to select the 20 best images in each part, neutral and pleasant landscapes. To choose the images with the best averages in pleasure and the lowest delta per subject, we followed these steps to choose pleasant landscape images: (i) calculation of pleasure averages per image: we calculated the average pleasure scores given to each image by all subjects; (ii) selection of images with the best averages: we identified images with the highest pleasure averages, indicating they were generally preferred by subjects; (iii) calculation of delta per subject: for each subject, we calculated the difference between their pleasure score for each image and the general average pleasure for that image to assess individual variation in responses; (iv) selection of lowest deltas: We identified images for which the average delta per subject was lowest, suggesting greater consistency in subject responses for these images.

The final selection comprised 20 neutral and 20 pleasant landscape images, chosen for their effectiveness in evoking pleasure motivation [see examples of neutral image (a) and pleasant landscape image (b) in Supplementary Figure S1]. Pleasant landscape images had a mean pleasure rating of 8.2 out of 10, while neutral images averaged 3.6.

### 2.3 Data collection

#### 2.3.1 Experimental paradigm

The experimental paradigm consisted of two experimental blocks. The first block, which was necessarily at the beginning of the experiment, involved *passive observation*. The second block involved *active observation*, where participants were asked to imagine themselves in the scene. It was not possible to randomize the order of these two blocks because if participants became aware of active observation, they were likely to imagine themselves in all other scenes without being able to return to a state of passive observation.

In each block, we had 10 pleasant landscape pictures and 10 neutral landscape images mixed and presented randomly. In the middle of each block, there was a 2-min break. This applied to both the active and passive blocks. E-Prime software (Psychology Software Tools, Inc., Pittsburgh, PA, USA) was used for randomized stimulus presentation with specific durations (10s for image display, 8 s interstimulus interval, and 2 s fixation cross).

The experimental trial design was based on established research practices and our team’s previous studies (Lelard et al., 2013; Lelard et al., 2017; Vonesch et al., 2023; Akounach et al., 2022; Beaumont et al., 2021). We chose a 10-s image presentation time, which is optimal for accurate posturography recordings and allows for the development of clear emotional responses. The interstimulus interval was designed to mitigate carry-over effects and allow proper recording of postural and physiological measures, including skin conductance. This design ensures adequate time for emotional responses to manifest and physiological parameters to stabilize between stimuli, as supported by literature in the field (Stins and Beek, 2007; Boucsein, 2012).

After completing the postural and physiological data recording session and a short break, participants proceeded to a separate rating stage. For this final phase, each participant was seated in front of the screen, equipped with headphones and a miniature keyboard. The images are shown in random order, with each image displayed for 3 s, followed by 5 questions: On a scale from 1 to 9, to what extent do you find this image pleasant, unpleasant, approachable, avoidable, and intense?

The questions were heard through the headphones, and at the bottom of the screen, a scale from 1 to 9 was displayed to illustrate the range, with labels such as “very pleasant” on one end and “not at all pleasant” on the other to facilitate understanding. Participants were instructed to respond as quickly as possible using the keyboard, pressing a number from 1 to 9 for each question. The experiment automatically moved to the next image after all questions were answered.

#### 2.3.2 Posturography and electrodermal activity

Images were displayed on a screen placed 1.20 meters from the posturographic platform. This distance was carefully selected based on (i) experimental considerations with previous studies having consistently adopted distances ranging from 1.20 to 1.40 meters (Vonesch et al., 2023; Stins and Beek, 2007; Horslen and Carpenter, 2011); (ii) theoretical considerations following, for example, E.T. Hall’s proxemics theory (Hall et al., 1968) with distances less than 60 cm generally perceived as “intimate” by humans and therefore 1.2 m avoiding to create uncomfortable proximity for participants, which could potentially influence their responses or behavior during the experiment. To further validate this setup, we always asked participants to confirm that they have an optimal view of the screen before proceeding with the experiment.

A synchronization signal was sent to a Biopac MP150 system via Acqknowledge (Biopac Inc., Goleta, CA, USA software). An AMTI posturographic platform was connected to record postural measures, particularly the displacement of the Center Of Pressure (COP) in the anteroposterior and mediolateral axes.

Additionally, electrodes placed on the fingertips of the non-dominant hand recorded electrodermal activity during visualization.

Both signals were acquired at 1000 Hz. Posturographic data were downsampled to 100 Hz and low-pass filtered at 5 Hz with a 2nd-order Butterworth filter to eliminate unwanted frequencies. The EDA signal was filtered with a cut-off frequency of 5 Hz to eliminate high-frequency noise and downsampled to 10 Hz. Ledalab (a MATLAB toolbox) was subsequently used to analyze the signal and extract the phasic component.

#### 2.3.3 Psychometric data

To investigate the relationship between individual characteristics and physiological responses, we administered a battery of psychometric instruments after completing all experimental tasks. This timing ensured that the questionnaires did not influence participants’ responses during the main experimental tasks. These included French-validated versions of four widely recognized questionnaires. The Inclusion of Nature in Self (INS) scale (Schultz, 2001) consisted of seven pairs of circles, one labeled “me” and the other “nature,” which overlapped to varying degrees. The pairs ranged from two completely separate circles to two almost entirely overlapping circles. Each pair of circles corresponded to a number (1 for completely separate circles to 7 for the most overlapped). A high score represented a strong inclusion of nature in the self. This single-item measure asked respondents to choose the pair of circles that best represented their sense of connection to the natural world. The INS has been designed to assess an individual’s cognitive representation of their relationship with nature at an abstract level.

The Connectedness to Nature Scale (CNS) focused on the relationship with nature from a predominantly cognitive and experience-based perspective. This scale had a unifactorial structure. In our study, we used a short version of the scale (Mayer and Frantz, 2004) consisting of 10 items, with a 7-point Likert-type response scale (ranging from 1 “Strongly disagree” to 7 “Strongly agree,” with 4 as “Neutral”). The CNS measured an individual’s subjective cognitive connection to nature, assessing the degree to which people feel part of the natural world. This scale had been widely used in environmental psychology research and had shown good reliability and validity in various studies (Navarro et al., 2017).

The third was a multidimensional empathy scale (Interpersonal Reactivity Index) created by Gilet et al. (2013), which quantified various components of empathy.

Finally, we utilized a comprehensive personality inventory (Bigfive Inventory) that focused on traits associated with nature affinity, as outlined by Courtois et al. (2020).

In addition to demographic data such as age and gender, participants were asked to provide information about their place of residence (rural or urban) and the frequency of their outings, which was assessed using a scale ranging from 1 (rarely) to 5 (very frequently).

#### 2.4 Statistical analyses

Data normality was assessed using the Shapiro–Wilk test and visual inspection of boxplots. Non-normal distribution led to the application of non-parametric methods. For all analyses involving postural or physiological data, the Friedman test was chosen to compare four groups. This test was used to analyze differences in the primary dependent measures (postural or physiological variables such as center of pressure displacement, standard deviation, path length, or electrodermal activity). The independent factors in these analyses were the image types (pleasant landscapes vs. neutral landscapes) and the observation conditions (active vs. passive). For ratings analyses, the Wilcoxon signed-rank test was conducted to compare the two paired groups. Post-hoc analyses were conducted to examine specific group contrasts following the primary statistical tests. Given the non-parametric nature of the data and the need to control for multiple comparisons, the Nemenyi test was selected. This method is particularly appropriate for pairwise comparisons when using rank-based tests such as Friedman’s ANOVA, as it accounts for the tied ranks and ensures robust control of Type I error rates. Correlations were evaluated using Spearman’s rank coefficient, yielding both rho (*ρ*) values and associated *p*-values. All analyses were performed in R (R Core Team, 2024), with statistical significance set at *p* < 0.05.

## 3 Results

### 3.1 Ratings

**Figure 1:**
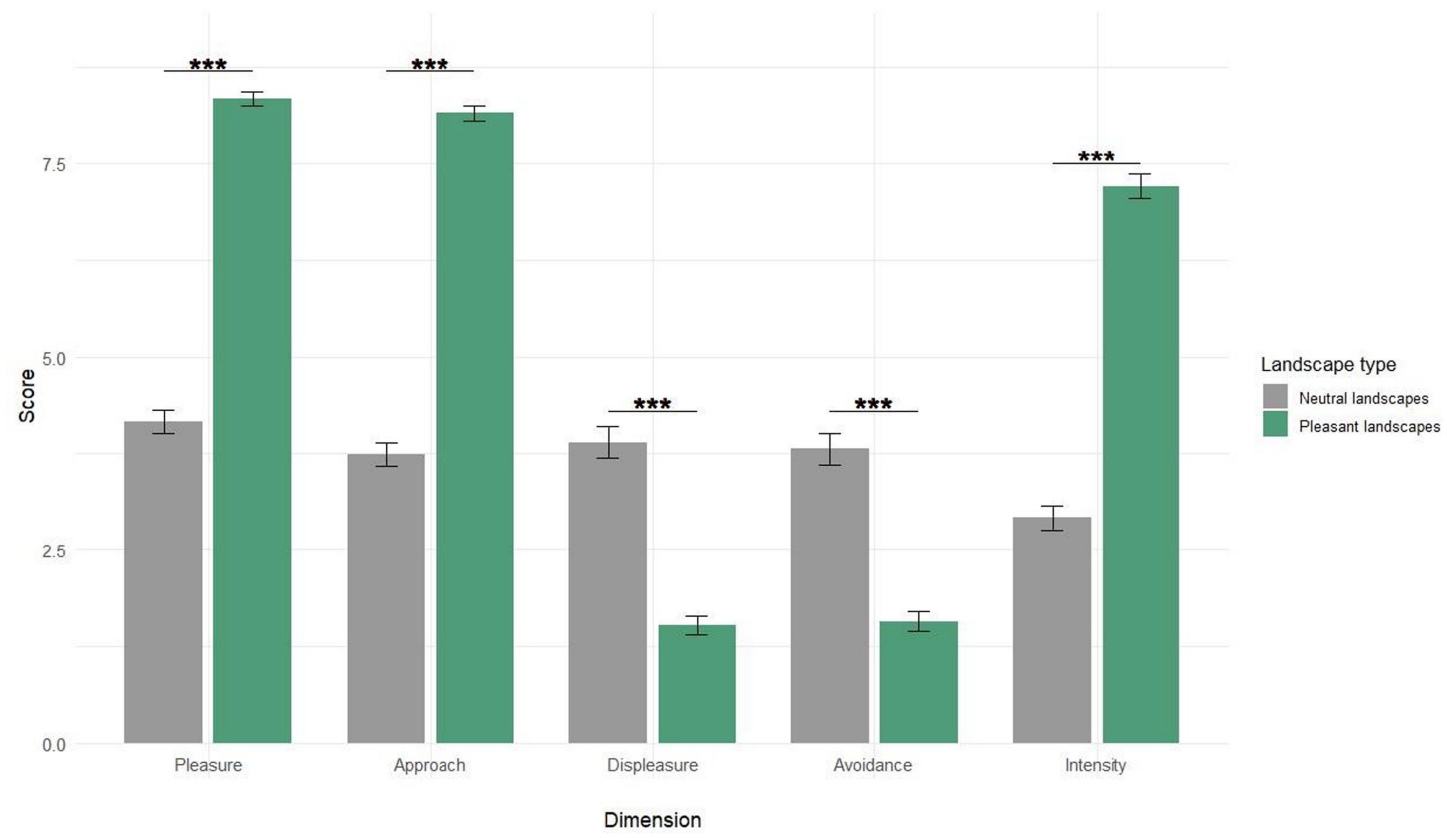
Mean (±SEM) subjective responses to pleasant vs. neutral landscapes. Ratings are shown on a 1-to-9 scale for *Pleasure*, *Displeasure*, *Approach*, *Avoidance*, and *Intensity* of emotional response to the presented images. *** Means *p* < 0.001.

Participants’ subjective responses (Figure 1) demonstrated statistically significant distinctions between the two landscape categories. Among all the rating dimensions, we chose to highlight *Pleasure* in the results. Statistical analysis using the Wilcoxon signed-rank test (*V* = 238,032, *p*-value <2.2e-16) revealed a marked difference in pleasure ratings between neutral (4.16 ± 1.72) and pleasant landscapes (8.34 ± 1.05). This pattern of differentiation was consistent across all measured dimensions (*p* < 0.001). These results confirm the effective categorization of the visual stimuli.

### 3.2 Postural responses

**Figure 2:**
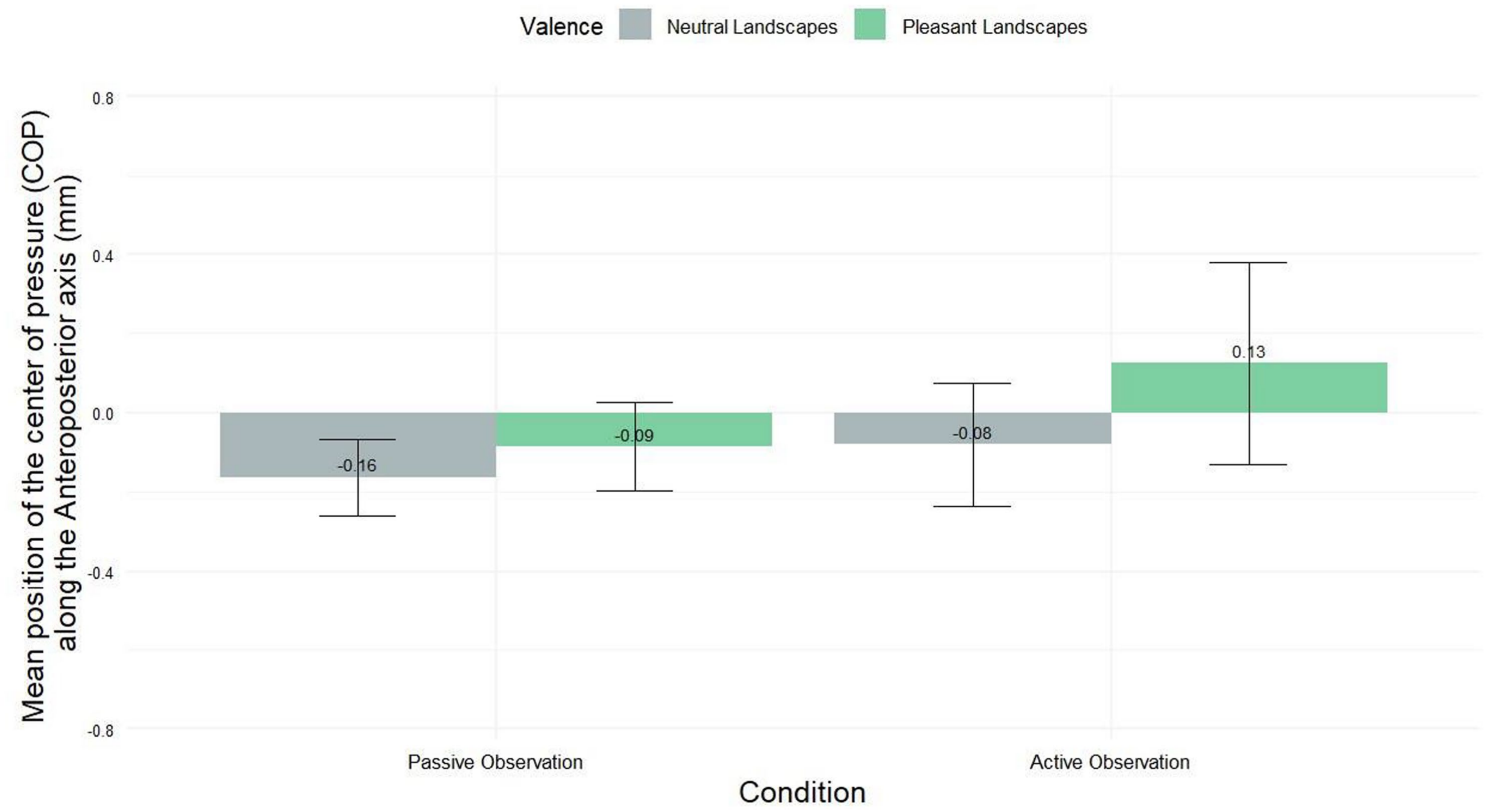
COP’s displacement (mm) in the anteroposterior direction for pleasant and neutral landscapes across active and passive viewing conditions (mean ± SEM).

Figure 2 illustrates differences in the displacement of the center of pressure along the anteroposterior axis. Although not statistically significant, a notable shift in postural behavior was observed between passive and active viewing conditions. During passive observation, participants exhibited a slight avoidance tendency (−0.09 mm ± 1.00). In contrast, active observation elicited an approach-like behavior (0.13 mm ± 2.42). Statistical analysis using the Friedman test (χ^2^ = 4.9886, df = 3, *p*-value = 0.1726) did not reveal significant differences between the four conditions. However, this transition from minimal avoidance to approach suggests a potential influence of engagement level on postural responses to visual stimuli.

**Figure 3:**
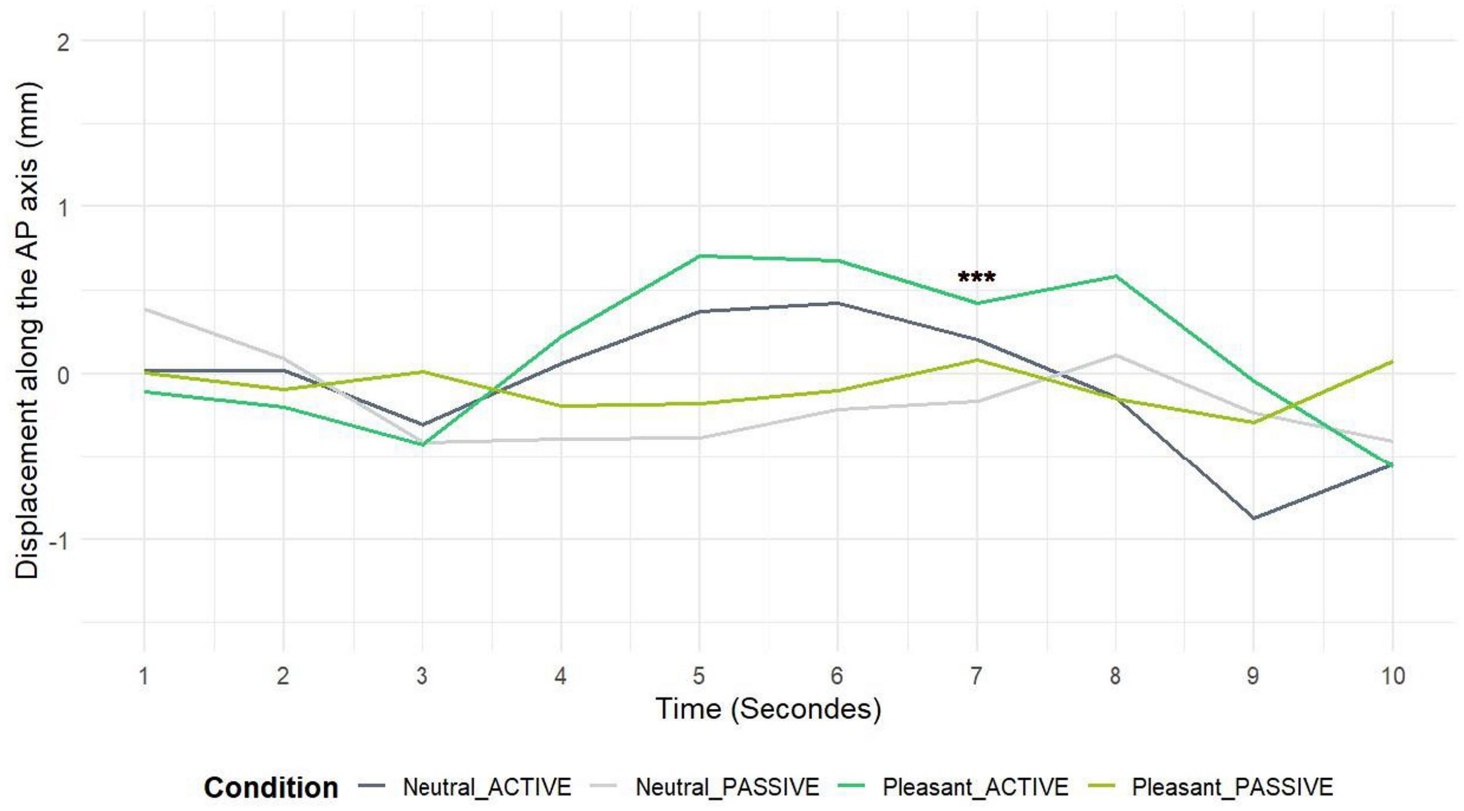
Time course over 10 s of observation for pleasant vs. neutral landscapes. The figure shows changes over time during both active and passive observation conditions. *** Means *p* < 0.005.

To better understand this approach behavior, the temporal course across all seconds of visualization was established for both observation conditions (see Figure 3). To avoid Type I errors due to multiple comparisons, we corrected the *p*-values by dividing the significance threshold (0.05) by the number of factors (in this case, 10), resulting in a threshold of 0.005. This ensures that our conclusions are robust against the risk of false positives.

A significant difference was observed at second 7. Statistical analysis using the Friedman test (χ^2^ = 16.55, df = 3, *p*-value = 0.00087) confirmed this difference, indicating a statistically significant variation among the four conditions at this time point. Post-hoc tests revealed notable variations between conditions. For active observation, the mean for neutral landscapes was 0.2 mm, while pleasant landscapes reached 0.42 mm. Regarding passive observation, the means were −0.17 mm for neutral landscapes and 0.08 mm for pleasant landscapes, respectively. The tests revealed significant differences between pleasant and neutral landscapes in active (*p* = 0.0001) observation condition. This suggests a particular approach toward pleasant landscapes, especially evident in active observation, thus highlighting the importance of embodiment and immersion in these visual experiences.

Initially, no significant effects were found when postural indices were averaged over several seconds. Therefore, we further examined the temporal dynamics by analyzing the posturographic responses for each second of exposure. This analysis revealed the significant difference at second 7, which was further confirmed by post-hoc tests.

**Figure 4:**
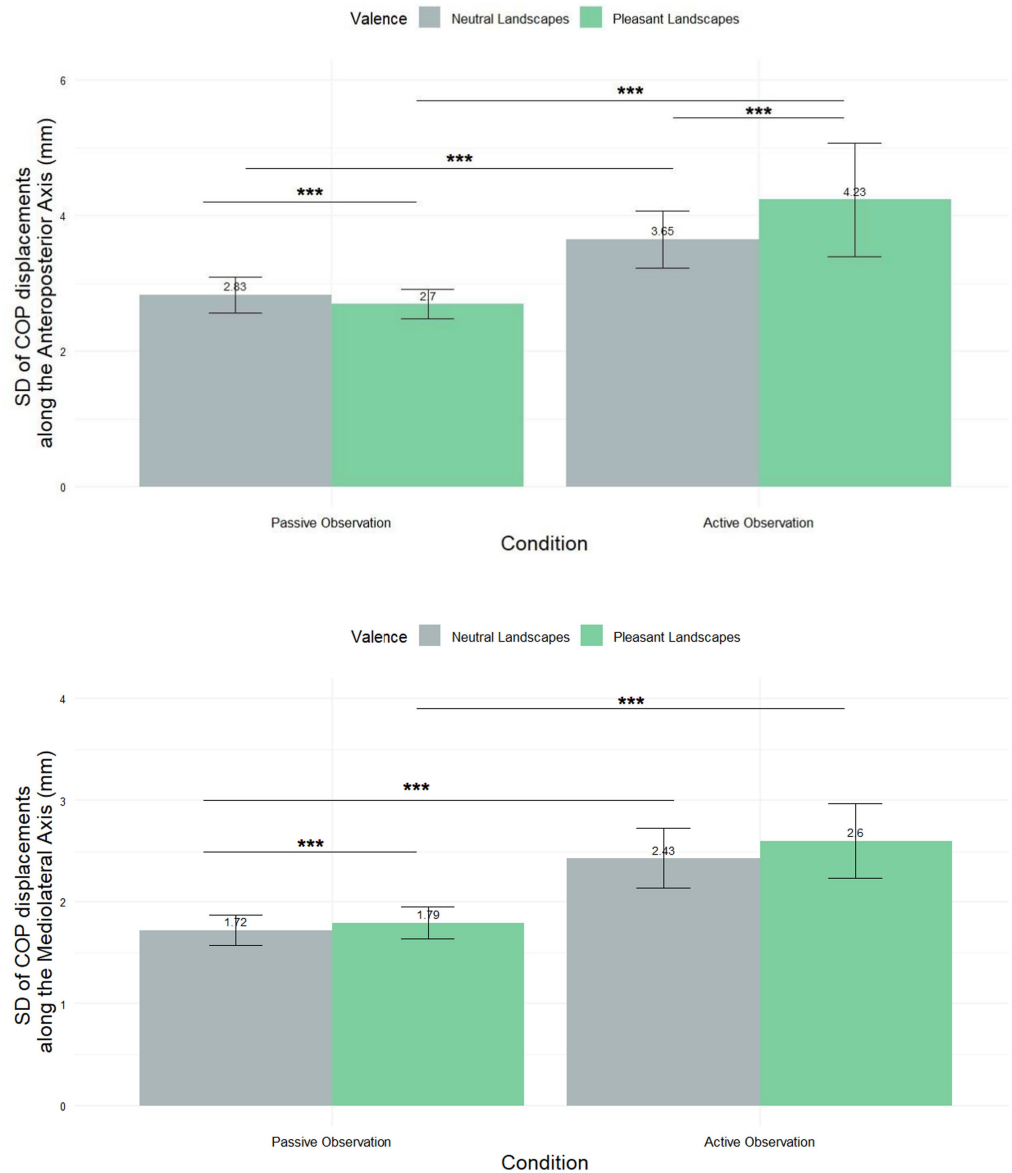
Mean (±SEM) standard displacement of the COP in the anteroposterior direction (SD [COP]-AP) and in the mediolateral direction (SD [COP]-ML). *** Means *p* < 0.001.

In addition to analyzing the mean displacement of the center of pressure (CoP), we calculated the standard deviation to assess the variability of postural adjustments across conditions. This measure provides insights into the stability and adaptability of postural responses, complementing the information derived from mean values Figure 4A. A shows a significant difference in SD [COP-AP] across conditions. Statistical analysis using the Friedman test (χ^2^ = 17.331, df = 3, *p*-value = 0.0006) confirmed this difference. For passive observation, the mean for neutral landscapes was 2.83 ± 2.5, and for pleasant landscapes it was 2.7 ± 2.05. In active observation, the mean for neutral landscapes was 3.65 ± 4.0 and for pleasant landscapes, it was 4.23 ± 7.99. *Post hoc* tests revealed significant differences between all groups, with *p* < 0.001, further highlighting the distinct responses elicited under each condition.

In the mediolateral axis (Figure 4B), a Friedman test (χ^2^ = 17.331, df = 3, *p*-value = 0.0006) revealed differences in SD [COP-ML]. For passive observation, the mean for neutral landscapes was 1.72 mm ± 1.44 and for pleasant landscapes, it was 1.79 mm ± 1.49. In active observation, the mean for neutral landscapes was 2.43 mm ± 2.81 and for pleasant landscapes, it was 2.6 mm ± 3.54. Post-hoc tests revealed no significant difference between Neutral_ACTIVE and Pleasant_ACTIVE conditions, while significant differences were observed between other condition pairs (*p* < 0.001).

**Figure 5:**
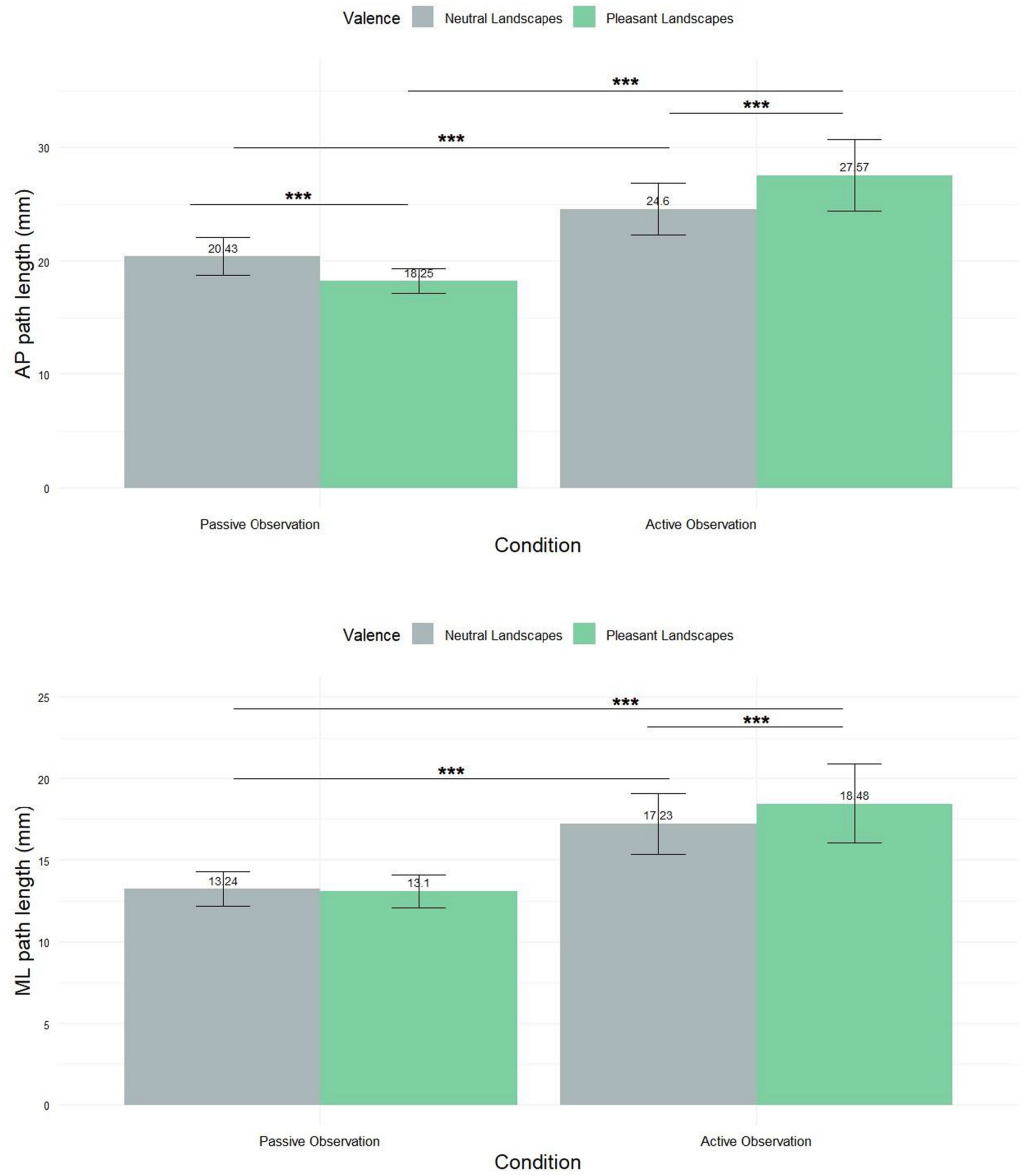
Mean (±SEM) path of COP displacement along the anteroposterior axis (AP) and the mediolateral axis (ML). *** Means *p* < 0.001.

A Friedman test revealed significant differences in path length across conditions in the AP axis (Figure 5A) (χ^2^ = 30.051, df = 3, *p*-value = 1.34^e^-06). In passive observation, the mean path length for neutral landscapes was 20.43 mm ± 15.72, and for pleasant landscapes 18.25 mm ± 10.31. In active observation, neutral landscapes yielded 24.6 mm ± 21.72, and pleasant landscapes 27.57 mm ± 29.72. Subsequent pairwise comparisons unveiled statistically significant variations across all experimental groups, with *p*-values consistently below 0.001.

For the mediolateral axis (Figure 5B), the Friedman test revealed significant differences (χ^2^ = 13.663, df = 3, *p*-value = 0.003402). *Post-hoc* tests revealed significant differences between all group combinations (*p* < 0.001) except for the comparison between neutral and pleasant landscapes in the passive condition. In passive observation, neutral landscapes showed a mean of 13.24 mm ± 10.08, and pleasant landscapes 13.1 mm ± 9.56. Active observation yielded 17.23 mm ± 17.84 for neutral and 18.48 mm ± 23.2 for pleasant landscapes.

Statistical analysis of electrodermal activity was conducted using the Friedman test (χ^2^ = 0.49091, df = 3, *p*-value = 0.9209), which did not reveal significant differences between conditions. However, it is important to note the observed response trends. In the passive observation condition, neutral landscapes elicited a mean response of 0.36 μS ± 0.64, while pleasant landscapes yielded 0.34 μS ± 0.56. Active observation showed higher mean responses: 0.54 μS ± 1.25 for neutral landscapes and 0.56 μS ± 1.29 for pleasant landscapes. Although not statistically significant, these trends suggest a potential increase in physiological arousal during active observation compared to passive viewing.

### 3.3 Correlations

We calculated correlations between participants’ psychometric measures and certain scores derived from questions posed to them. Specifically, we included the Outing Nature score, which reflects the frequency of nature outings based on participants’ responses, as well as other psychometric measures such as the CNS (nature connectivity score), INS (inclusion of nature in self score), IRI (interpersonal reactivity index score), and its components: COG_Emp (cognitive empathy) and EMP_Emp (emotional empathy). Additionally, personality traits from the Big Five questionnaire were considered, including Agre (agreeableness) and Open (openness to experience).

For the correlation analysis, we focused on the delta (*Δ*) of all posturographic and physiological measures. The delta represents the difference between values obtained for pleasant and neutral landscapes for each measure, under both passive and active observation conditions. These measures include the displacement of the center of pressure (COP), standard deviation (SD), and path length (LengthPath).

The analysis revealed several significant correlations (see Supplementary Table S1). A negative correlation (rho = −0.47, *p* < 0.01) was found between the delta of the center of pressure displacement and the openness personality trait from the Big Five questionnaire. Additionally, strong negative correlations were observed between the Outing Nature score and both ΔSD [COP-AP] and ΔLength-AP Pass (rho = −0.71 and −0.61, respectively, *p* < 0.001). These findings suggest potential links between personality traits, engagement with nature, and postural responses to visual stimuli.

## 4 Discussion

Our study is part of the emerging and fascinating field of environmental neuroscience. Within this field, it pioneers the use of posturography to study the motor and postural responses induced by the perception of pleasant versus neutral environmental landscapes/scenes. This study is in continuity with previous studies in which we showed the importance of emotions in the psychological processes involved in the perception of visual pollution (Beaumont et al., 2020) and preliminary results on the postural responses induced by this perception.

Regarding subjective data, our results confirmed that Pleasant landscapes evoked significantly higher *Pleasure*, *Approach*, *Intensity,* and lower *Displeasure* and *Avoidance* than Neutral Landscapes. Firstly, these results validated the selection of the stimuli made among the larger database to select the most adapted visual scenes for the “pleasant landscapes” and “neutral landscapes” experimental conditions. These results are following those of Beaumont et al. (2020) and Akounach et al. (2022), where clean environmental scenes evoked higher pleasure and approach desire than polluted ones. In the scientific literature, a large number of studies have focused on the subjective feelings and emotions induced by exposure to natural scenes. Mainly, the fact that pleasant landscapes induce higher ratings in the two dimensions generally taken into account in emotion theories (valence–here, positive–and intensity/arousal) corroborates the central and early role of emotions postulated in biophilia theory (Ulrich, 1983). The higher *Approach* and lower *Avoidance* ratings induced by pleasant landscapes refer to the “Motivation or Action Impulse” also postulated by the biophilia theory, for example, referring to a more or less innate (then early) tendency to seek connection with natural scenes (Ulrich, 1983). Our results also support this central place. Recently, Schiebel et al. (2022) demonstrated, using different classical psychological tasks testing automatic approach tendencies, a clear effect of nature: through psychometric indexes and performances, a clear automatic approach tendency appears in humans.

As the literature on the effect of exposure to natural/pleasurable scenes on the brain is vast, the question arises as to the specific activation of brain circuits corresponding to those motor and/or emotional dimensions that appear central to psychological theories of environmental perception. As explained in our introduction, functional neuroimaging investigation of the neural correlates of exposure to natural scenes/landscapes does not appear to be fully capable of capturing the neural circuits associated with the subjective impressions reported here, even though several studies point in the direction of activation of these circuits, underlining their importance in the aesthetic aspect of judging natural scenes. Again, for the emotional dimension, the functional neuroimaging literature is quite different. Indeed, the vast majority of studies that have investigated neural responses to exposure to natural scenes/landscapes have reported preferential activation of emotional circuits (see our introduction).

To our knowledge, this study is the first one to apply posturography to the framework of biological processes involved in nature/landscapes perception. More precisely, through the lens of theoretical and experimental studies showing the importance of emotional processes in landscape/nature appraisal, our purpose was to compare responses to landscapes judged as “pleasant” and ones judged as “neutral.” Regarding the mean postural responses recorded in the different experimental conditions, we report a non-significant modulation of the COP-AP whereas the SD and Path Length of COP’s displacements were significantly modulated by the valence (pleasant vs. neutral) and simulation (passive vs. active observation) factors. Our results demonstrate a slight shift for pleasant landscapes from a slight withdrawal during passive observation to a slight approach-type behavior during active observation (Figure 2). This shift was not reported for neutral landscapes, for which a light withdrawal was reported both in the passive and in the active conditions. Recently, the modulation of postural control by the simulation factor (when going from passive to active viewing) was observed for high and low painful stimuli perception (approach becoming avoidance; Beaumont et al., 2021) and for polluted environmental scenes perception (approach becoming avoidance; Akounach et al., under review). In accordance with previous studies, this postural shift does not correspond to a subjective shift, as, here, pleasant landscapes have induced significantly higher levels of *Approach* and lower levels of *Avoidance* as compared to neutral landscapes, underlying a sort of dichotomy between subjectively reported (rating obtained through Likert scales) and objectively measured (through posturography) approach-avoidance tendencies. These effects have been extensively discussed in the framework of painful stimuli perception (Beaumont et al., 2021). Briefly, we argued for an effect of consciousness deployed in time: early posturographic responses are mainly related to automatic control processes, becoming, along time and under the influence of mental simulation, more “conscious” and being at the foreground of a possible inversion in time of the approach-avoidance behavior.

The lack of statistical significance between the different experimental conditions on the COP-AP and COP-ML variables is also noted in previous research on other functional contexts such as empathy for pain (Lelard et al., 2017, 2013) or pollution perception (Akounach et al., under review). However, in order to gain a better understanding of the postural correlates of visual scene processing (and possibly of associated socioaffective processes), recent studies (Lelard et al., 2017; Mouras and Lelard, 2018) have shown the importance of analyzing the temporal dynamics of postural cues over the entire duration of visual stimulus presentation. Within the framework of painful stimuli perception and using this temporal dynamic extraction approach, we reported a posterior displacement at different times during stimulus presentation (4 s; 9–12 s) of the COP in response to active viewing (i.e., mental simulation) of painful as compared to non-painful stimuli. Through a similar analysis approach, we report here (3) significant effects at the 7^th^ second of the stimulus presentation with an avoidance behavior in the Neutral-Active condition echoing three approach behaviors of increasing intensity for Pleasant-Passive, Neutral-Active, and Pleasant-Active conditions. While the mean effects are not significant, these results show that the factors of landscape valence (pleasant vs. neutral) and mental simulation (passive vs. active vision; stimulating the participant’s engagement with the visual scene, scene embodiment) have an impact.

While the mean effects are not significant, these results show that the factors of landscape valence (pleasant vs. neutral) and mental simulation (passive vs. active vision; stimulating the participant’s engagement in the visual scene, scene embodiment) induce a significant tendency to approach precisely at the 7th second of viewing: in passive vision, we get significantly closer to a pleasant landscape than to a neutral one; in active vision, we also get closer to a pleasant landscape than to a neutral one. With this 7-s time point anchored in mind, it seems reasonable to look at the temporal evolution of the COP’s position over the entire 10-s presentation of images belonging to the different experimental conditions 3. Looking at the time course as a whole, it seems reasonable to say that the period of 7 s after the start of the scene presentation seems to be the culmination of an embodiment/mental simulation effect beginning at around 3 s and inducing the transition from a slight postural withdrawal (which can be noted in all experimental conditions) to a more marked approach notable in the 3 experimental conditions: Neutral-Active, Pleasant Landscapes-Passive, and Pleasant Landscapes-Active. At 7 s and in the passive condition, there was a more pronounced approach to pleasant landscapes than to neutral ones.

An important question is the timing at which this effect manifests itself. Whereas in passive vision, the landscape valence effect seems to be exerted moderately but relatively “early” (during the first 4 s of landscape vision) and without exerting any particular inflection on the “later” temporal course, the “embodiment” effect, interpreted as reflecting the subjects’ immersion, seems to be central in the appreciation of landscapes and “later” in its influence on postural responses to landscapes. Once again, this study is the first to use posturography to investigate motor processes related to the perception and appreciation of pleasant natural scenes. As a result, our interpretations are based on a corpus of data that will obviously have to be replicated, but they seem compatible with previous results obtained in other functional contexts and theories pertaining notably to environmental psychology that may resonate with these findings:

- the pertinence of posturography to capture biomarkers of the motor and affective correlates of pleasant landscapes appraisal as compared to other techniques such as neuroimaging where activations within the motor circuits are not often reported and, even when reported, do not translate into a real activation of a motor behavior or other physiological responses such as galvanic skin response etc.
- the confirmation of the essential role of the interaction between motor and affective responses for nature/landscapes appreciation which has been theorized by several important environmental psychology theories (see Yan et al., 2023 for a review of theories of “landscape preferences”);
- the demonstration of the involvement and importance of the subject’s immersion (and successful embodiment of the visual scene processed), which seems to unfold over a relatively long and “late” period (compared with a “valence” effect). In our view, a very important question is the particular nature of the psychological and neural processes underlying the transition from passive to active vision, where the participant is askedto imagine being in the scene represented. Is this an effect on perceptive immersion, a motor-imagery effect in which the participant “projects” themselves into the scene, or a particular appeal to the participants’ ecological sensitivity and personal history, which certainly modulate their behavioral tendencies towards nature? Certainly, it is a little of all of this at once.

Results obtained on other mean postural indexes are also of major importance. As illustrated in Figures 4, 5, many significant differences have been found between the experimental conditions but cannot all be here discussed. Regarding the *mental simulation factor*, we found a higher SD-COP-AP, SD-COP-ML, AP-path length and ML-path length values in the active viewing condition as compared to the passive one. In recent research focusing on the posturographic correlates of pollution perception (Akounach et al., submitted), we reported the same results in response to a pollution factor (with a potentization of this effect by the mental simulation factor). Very importantly, these results first show the pertinence of the SD-COP displacement and the Path-Length as pertinent postural biomarkers of landscapes/natural scenes appraisal (in opposition to COP displacement *per se*).

When looking on the scientific literature, one can note: (i) in a postural threat condition, a lower AP-Path length in response to aversive stimuli (Lelard et al., 2014); (ii) a positive correlation between these indexes and the level of anxiety, suppressed when eyes were closed (Ohno et al., 2004); (iii) Doumas et al. (2018) demonstrated an additive effect of stress and time pressure on sway amplitude. Through the lens of these results, we would interpret this as the posturographic correlate of the embodiment process mentioned above and induced by the active vision task. In some sense, this embodiment/incarnation process is reflected by a certain postural instability that could be important to interconnect with the co-occurring emotional processes. This interconnection between emotion and postural instability could be one of the biological signatures of embodiment. This interpretation has to be articulated with the results regarding the *landscape valence* factor: within the passive condition, viewing pleasant landscapes induced, in the anteroposterior dimension, significantly lower SD-COP’s displacement and Path length. These results are opposite to those obtained in response to polluted environmental scenes (Akounach et al., under review) which increased these postural indexes. They can be interpreted as a sign of increased postural stability in response to pleasant landscapes as compared to neutral ones being viewed. These results support important theoretical aspects of numerous theories of nature exposure benefits [see for example Yan et al. (2023); Berman et al. (2021) for a review of these different theories] that have in common the proposal of the same kind of effect involving less postural control. Obviously, the kind of experimental paradigm proposed in this pioneer study is not adapted to precisely discriminate one theory from another one. The precise manipulation of the theorized processes in each of these theories is certainly a very interesting research agenda. One interesting direction that could be used to specify the link between the postural responses and precise psychological processes or traits is the correlational analysis between psychometric measures and posturographic responses. Here, we demonstrate negative correlations between posturographic measures and certain psychometric measures. This approach has to be considerably developed in future studies.

## Data Availability

The raw data supporting the conclusions of this article will be made available by the authors, without undue reservation.
