# Supplementary material for: Postural correlates of pleasant landscapes visual perception
Source: Front Psychol. 2025 Feb 5;16:1527691. doi: 10.3389/fpsyg.2025.1527691 (PMC11837975; doi:10.3389/fpsyg.2025.1527691)
Supplement: Supplementary file 1 [file Data_Sheet_1.DOCX]

a.
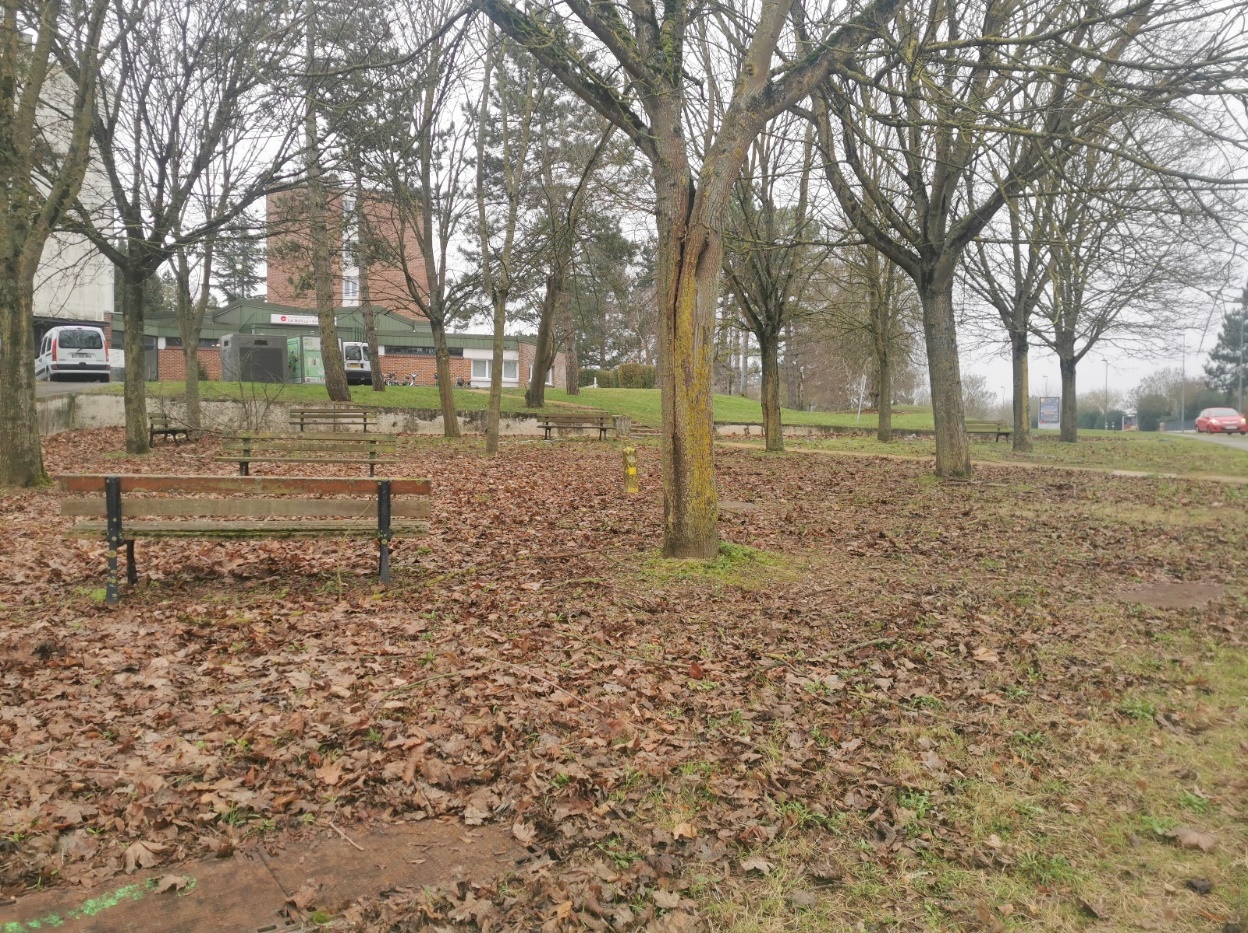


b.
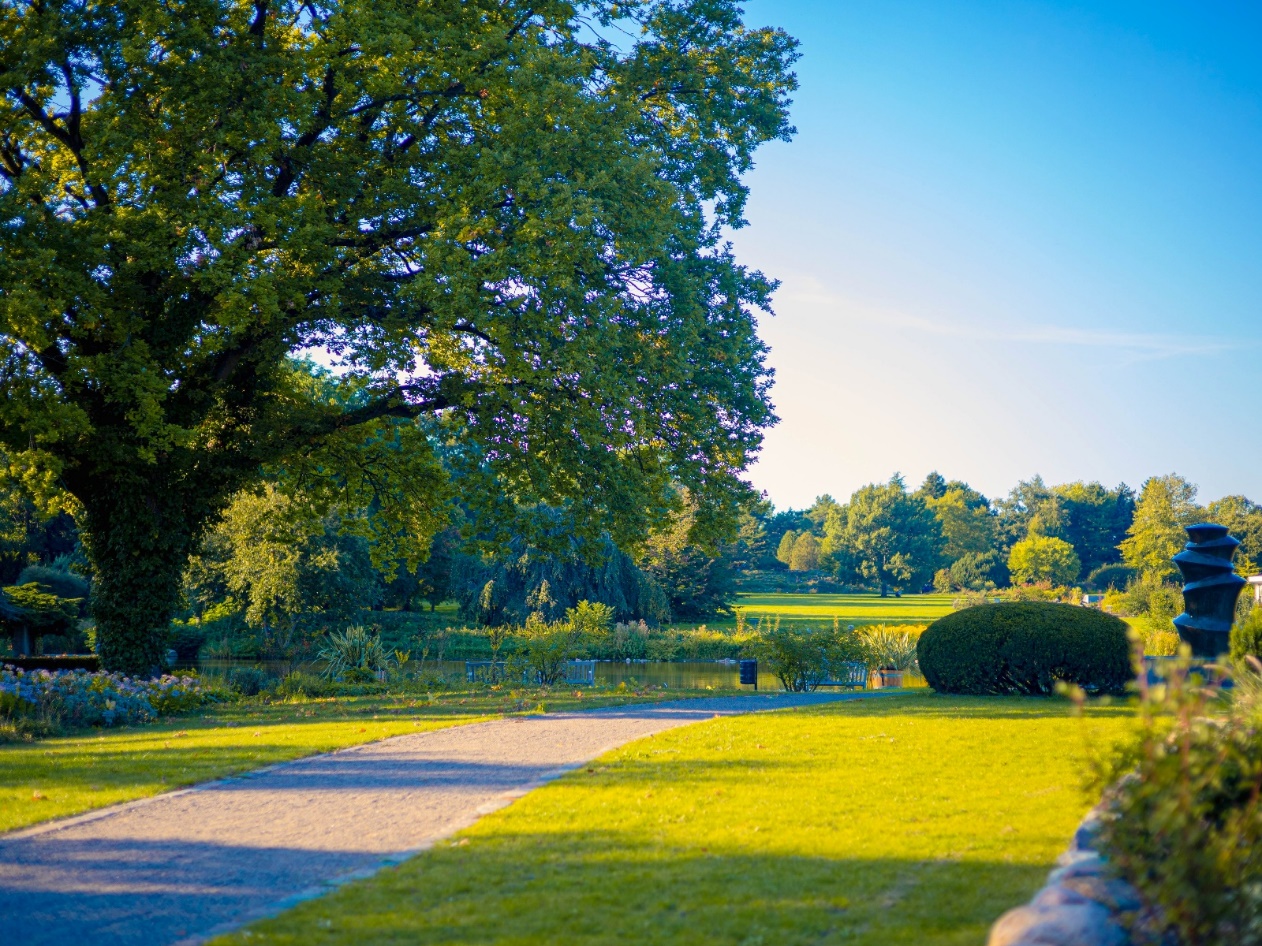


Figure 1 : Example of an image classified as a neutral landscape (a) and pleasant landscapes (b)


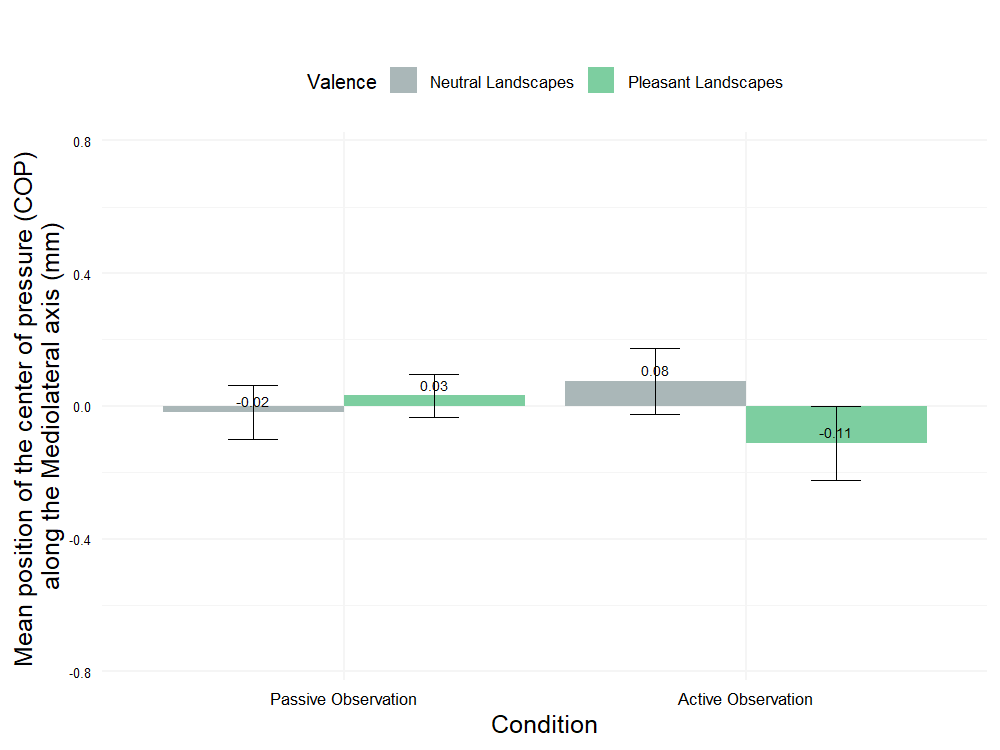


Figure 2 : COP’s displacement (mm) in the mediolateral direction for pleasant and neutral landscapes across active and passive viewing conditions (mean ± SEM).

Figure 2 illustrates the displacement of the center of pressure (COP) along the mediolateral axis (ML) under different conditions. The mean values (m) and standard deviations (SD) for the various conditions are as follows: for **PASSIVE observation**: Neutral Landscape (NeutrePay), m = -0.02 with SD = 0.77; for **PASSIVE observation**: Pleasant Landscape (Paysage), m = 0.03 with SD = 0.62; for **ACTIVE observation**: Neutral Landscape (NeutrePay), m = 0.08 with SD = 0.95; and for **ACTIVE observation**: Pleasant Landscape (Paysage), m = -0.11 with SD = 1.06. Statistical analysis using the Friedman rank sum test (χ² = 1.2857, df = 3, p-value = 0.7325) did not reveal significant differences between the four conditions.

In previous studies, the investigation of approach and avoidance behavior primarily utilized the anteroposterior (AP) axis of the center of pressure (CoP) as a key indicator in posturography. The focus on the CoP-AP was due to its relevance in understanding these specific behaviors. However, for the path length and standard deviation measures, both the anteroposterior and mediolateral (ML) axes are important, as we aim to assess overall movement and instability, not just in the AP direction.

Table 1 : Correlation matrix showing the relationships between Psychometric Measures and Postural/Physiological Variables using Spearman’s rank-order coefficient


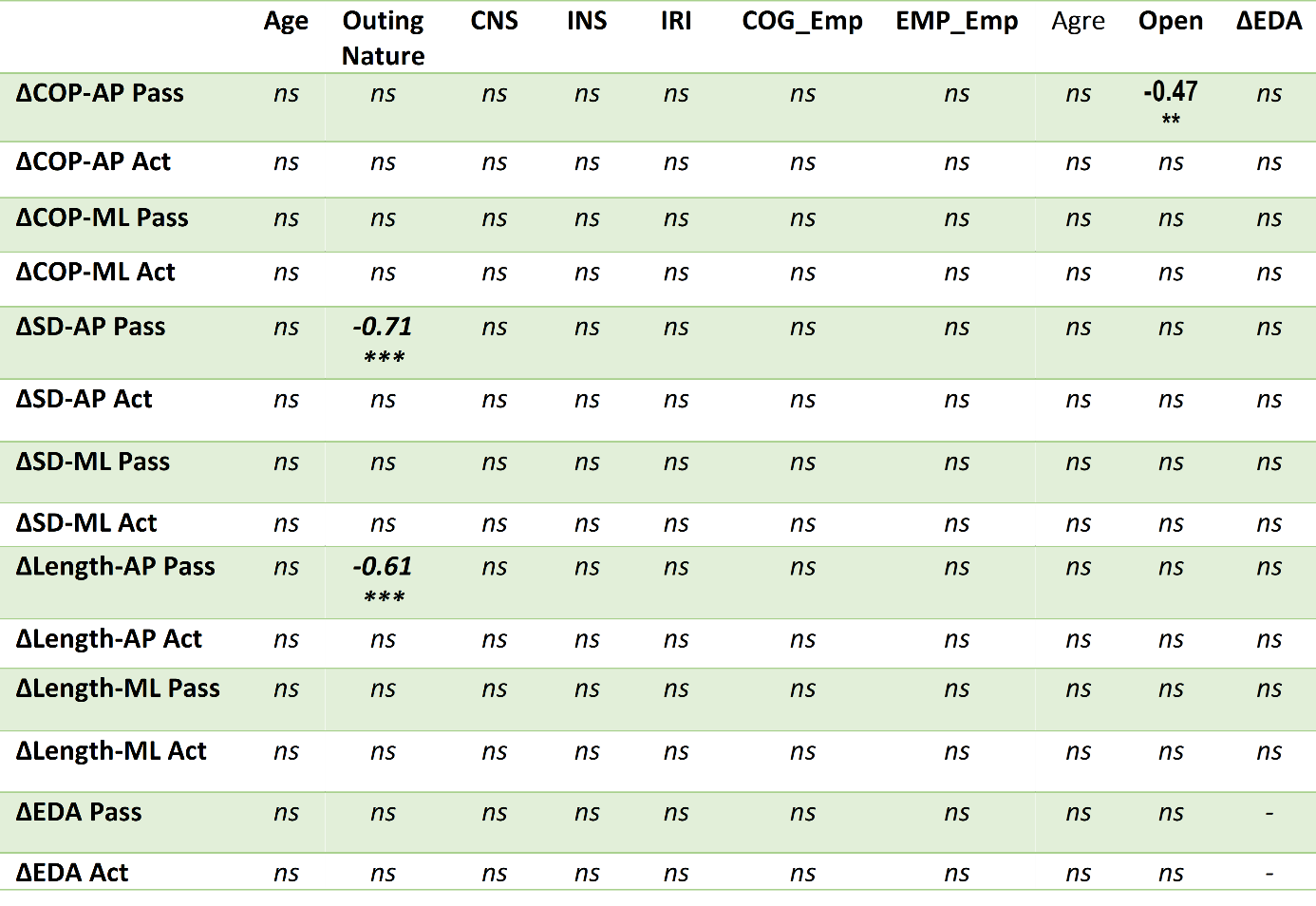


*Outing Nature: frequency of nature outings score; CNS: nature connectivity score; INS: inclusion of nature in self score; IRI: interpersonal reactivity index score; COG_Emp: cognitive empathy component from the IRI; EMP_Emp: emotional empathy component from the IRI; Agre: agreeableness score from the Big Five; Open: openness to experience score from the Big Five ; Δ: difference between values for pleasant and neutral landscapes for each measure In green: passive observation (Pass), and in light color: active observation (Act).ns: not significant, ** p<0.01, *** p<0.001.*
